# Difficulties and distress experienced by Japanese public health nurses specializing in quarantine services when dealing with COVID-19: A qualitative study in peri-urban municipality

**DOI:** 10.3934/publichealth.2023018

**Published:** 2023-04-14

**Authors:** Akari Miyazaki, Naoko Kumada Deguchi, Tomoko Omiya

**Affiliations:** 1 Graduate School of Comprehensive Human Science, Program in Nursing Science, University of Tsukuba, 1–1–1 Tennodai, Tsukuba, Ibaraki, 305–8575, Japan; 2 Faculty of Education, Shizuoka University, 836 Ohya, Suruga-ku, Shizuoka-shi, Shizuoka, 422–8529, Japan; 3 Faculty of Medicine, Division on Health Innovation and Nursing, Department of Public Health Nursing, University of Tsukuba, 1–1–1 Tennodai, Tsukuba-shi, Ibaraki, 305–8575, Japan

**Keywords:** COVID-19 pandemic, public health nurse, crisis communication, residents, resilient organization

## Abstract

Public health nurses (PHNs) are among the few municipal civil servants who lead community infection control and prevention initiatives in Japanese public health centers (PHCs). This study aims to investigate the distress faced by PHNs and clarify their difficulties and working environment relative to infection prevention control activities during the COVID-19 pandemic. We adopted a qualitative description methodology in this study of 12 PHNs who were involved in COVID-19 prevention and control in PHCs in Prefecture A. The distress during the early phase of the pandemic was due to the uncertainty of the SARS-CoV-2 related disease, which caused panic in medical institutions and among residents. PHNs were overwhelmed, distressed and exhausted by their inability to control the ‘pandemic’, lack of patient cooperation for prevention control and the unsustainable organizational environment. They were also distressed because they were one of the specialized personnel responsible for saving residents' lives with limited medical resources and while having identity crises due to an inability to carry out the PHN's role of controlling infection in the community. For future crises, rapid, drastic innovation defying conventional organizational systems is critical to reform sustainable organizations so that they play an effective role in the community. Innovation in crisis communication and strengthening the medical system will help achieve a resilient community in a health crisis.

## 1. Introduction

The coronavirus disease 2019 (COVID-19) pandemic spread throughout Japan after the first confirmed case in January 2020. The eighth wave is threatening people's lives and their social life, and, as of February 2023, >33 million confirmed cases, including 72,320 deaths, have been reported [1]. In Japan, public health nurses (PHNs) in public health centers (PHCs) represent the community frontline, as they are responsible for quarantine services during the pandemic.

The PHC is a municipal organization that has an essential role in promoting public health in Japan. Besides daily health activities, including mental health services, food hygiene and services for patients with intractable diseases, PHCs are expected to be the local health risk management base for coping with health crises, including food poisoning, infectious diseases, natural disasters and terrorism [2]. In the case of past natural disasters, including earthquakes, floods and nuclear incidents, PHCs have been playing roles such as visiting, consulting, health promotion and controlling medical resources. Since the Japanese national health system prioritized health issues with a focus more on non-communicable diseases than on infectious diseases, the number of PHCs was reduced to 468, which is a 45% reduction over 30 years [3]. Meanwhile, a worldwide pandemic occurred.

Japanese PHNs are nationally licensed nursing professionals, and those working in PHCs play the main role in quarantine services as the professional infection control workforce in the community. In Japan, medical examinations and diagnoses are held in local clinics and hospitals. While, PHCs carry out establishment and arrangement of the local medical system, consultation services, active epidemiological investigation of all confirmed cases, transporting patients and medical distribution, including tests and hospitalization [4], mainly by PHNs. Their heavy workload during the pandemic has been reported in the media [5],[6].

Although some studies have focused on medical care workers in hospitals during the pandemic [7]–[9], only a few studies have investigated public health workers' experience. About 60% of USA public health workers, including medical doctors, nurses, economists and local developers working for the Center for Disease Control and Prevention (CDC), local governments, and the university public health section, had a high burnout risk during the COVID-19 pandemic [10]. Also, about half of Chinese public health experts and municipal civil servants working for the CDC and local health sections had psychological distress [11]. In Japan, only a few studies have focused on PHNs during health crises including natural disasters. About 70% of PHNs dispatched to affected areas during the Great East Japan Earthquake experienced physical and mental disorders, including insomnia, fatigue and flashbacks [12]. After the flood disaster that occurred in 2009, PHNs reported experiencing much distress, as they were subjected to resident complaints, anger and unreasonable demands which did not align with their perception of their own role [13].

As no studies have reported Japanese PHNs' experience during an infection outbreak before the COVID-19 pandemic, their distress and difficulties during a pandemic are unknown. A few studies revealed that the PHNs who consulted at call centers during the first wave of the COVID-19 pandemic had difficulties such as “being a complaints counter instead of a health consultations provider” and “difficulty in triage test-applicable cases and arrangement of facilities for tests” [14]. About 27% of PHC staffs including PHNs have reported burnout [15]. These studies are largely limited to the early phase of the pandemic, and PHNs, especially those in charge of consultations. However, with multiple waves of COVID-19 epidemics, situations, medical systems and people's attitudes toward the pandemic have continued to change. PHNs are also likely to have experienced various difficulties and distress as the situation has changed. However, their experiences and feelings during the prolonged COVID-19 pandemic are unclear. No study has investigated the perceived difficulty, emotional distress, anger, anxiety, conflict and fear that PHNs have faced, nor the events and circumstances behind their distress throughout all phases of the pandemic. It is extremely important and urgent to improve public health systems, PHC organizational systems and medical resource preparedness in the event of emerging infectious diseases and disasters that are sure to arise in the future. The research question for this study is “what experiences have PHNs had during the COVID-19 pandemic”. Specifically, “what were their experiences of difficulty and distress, and how they felt” and, further, “what were the circumstances behind these experiences”.

This study aimed to capture the voices of Japanese PHNs specializing in quarantine services on the frontlines in communities during the long-term COVID-19 pandemic. We also focused on the difficulties and distress they experienced and clarified the background to these issues.

## 2. Materials and methods

### 2.1. Research design and participants

A qualitative description methodology explained by Sandelowski [16] was selected in our study. This study design is particular in terms of a rich and straight description of the subjects' experience, which stays close to the data without providing an in-depth conceptual description [17]. The qualitative descriptive methodology is suitable for use in studies aiming to explore experiences and phenomena which are not yet revealed, e.g., child educators' perspectives against risky play and nurses' beliefs and experiences about family presence during resuscitation [18],[19]. As we aimed to explore and describe PHNs' experiences during the pandemic which are not yet revealed, we chose this type of design.

Our study was conducted in Prefecture A, which is located in the Kanto area of Japan. Prefecture A is a peri-urban municipality which contains both urban and rural areas, and the number of COVID-19 confirmed cases tended to increase following after the Tokyo Metropolitan area. PHNs involved in COVID-19 prevention and control in PHCs at any point after January 2020 were recruited through leaflet distribution for all PHNs working in PHCs in Prefecture A. Those who belonged to organizations other than PHCs were excluded. We selected a variety of participants, considering their characteristics such as age, experience and their role in PHCs, until the data reached saturation.

### 2.2. Data collection and analysis

A qualitative descriptive study using semi-structured interviews was conducted to gain clear insight about the experiences of PHNs during the pandemic. One-on-one interviews were held either online or face-to-face from October to December 2021. An interviewer (AM) interviewed each participant once. The interviews were conducted in a closed room in PHC or in a meeting room in the university to protect privacy.

We asked questions to reveal the distress that PHNs experienced in their overall activities following the interview guide. The interview guide contained the open-ended questions: “Through all of your experience in response to the COVID-19 pandemic, what situation have you found most difficult or challenging?”, “What situation have you found difficult or challenging to deal with [community residents/ patients/ close contacts/ medical institutions/ central government office/ colleagues]?”, “Have you ever evoked the intention to resign? And, what were the triggering events or stressful situation?” For each experience and difficulty, their emotions, feelings, thoughts and mental and physical effects were explored in detail with ancillary questions. Additionally, to ensure clear and profound interpretation, follow-up questions were asked.

The dictated recording data were analyzed using inductive content analysis. A content analysis is defined as “a research technique for making replicable and valid inferences from texts to the contexts of their use” by Krippendorff [20], and it can be used on a broad level of abstraction [21]. We analyzed data by referring to the research procedure provided by Graneheim and Lundman [22]. We read the data several times to obtain the whole context and extracted the text about the PHN's distressful experiences and challenges, paying attention not to miss the contents and meaning of phrases. The text was divided and condensed into meaning units which were labeled with a code. Furthermore, categories were created considering the associations between codes in some dimensions focused on similarities and relations.

### 2.3. Rigor in analysis

In order to establish confidence in the results of this research, we ensured rigor by using the model of trustworthiness of qualitative research by Lincoln and Guba [23],[24]. Five components of trustworthiness were considered, namely, credibility, dependability, confirmability, transferability and authenticity.

To ensure credibility, member checking was conducted in the interview and in the process of data analysis to ensure accurate understanding and interpretation of the verbal data. During the analysis process, the data were discussed by a research group familiar with the topic. To ensure dependability, we avoided leading questions by using an interview guide with open-ended questions during the interviews. During the analysis process, three researchers confirmed the audit trail in the archives. As a result, we decided to ask the participants for an additional interview or to answer follow-up questions, if necessary. To ensure confirmability, two researchers checked the interpretation of raw data and extracted categories and their relationship to each other. We also referred to previous studies focusing on PHNs and PHCs to strengthen confirmability. To facilitate transferability of the results to the other researchers, we provided a detailed description of the phenomena and context of the PHNs' experience. To ensure authenticity, the interviews and analysis were conducted by researchers who were trained on qualitative interviewing techniques and analysis. Throughout the data analysis process, researchers conducted interpretation of the context of raw data and analysis with continuous reflection and self-criticism.

### 2.4. Ethical consideration

This study was approved by the medical ethics review board of the University of Tsukuba (Approval Number 1678, September 27, 2021). Participants were informed about the purpose, anonymity of collected data and right to withdraw participation. Participants were informed that the collected data would not be shared, but would be used exclusively for research purposes. Written consent was obtained from all participants.

## 3. Results

### 3.1. Samples

Twelve PHNs from six PHCs participated in the study (Table 1). They were all females; seven PHNs had <5 years of experience; two had 6–20 years of experience in middle management positions; three had >21 years of experience in management positions. Each interview lasted from 34 to 85 minutes. All participants participated in one interview once, and no participant needed an additional interview.

**Table 1.** Participant demographics.

No	Sex (M/F)	Age decade	Experience as PHN	Duration of interview
A	F	30 s	15 years	47 min
B	F	20 s	2 years	60 min
C	F	50 s	25 years	49 min
D	F	30 s	13 years	60 min
E	F	50 s	34 years	46 min
F	F	20 s	0.5 year	49 min
G	F	20 s	0.5 year	34 min
H	F	50 s	33 years	85 min
I	F	30 s	1 year	55 min
J	F	20 s	2 years	56 min
K	F	30 s	3 years	50 min
L	F	20 s	0.5 year	67 min

### 3.2. Structure

**Figure 1.**
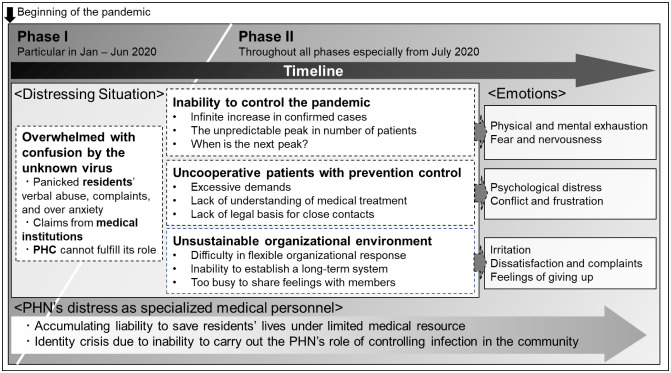
The structure of distressing situations, emotions and distress experienced by PHNs during the pandemic.

Figure 1 shows the structure of difficulties and distress experienced by PHNs. Their distress and background differed between the first phase of the pandemic, i.e., around January to June 2020 (Phase I), and the entire pandemic period, but especially from July 2020 (Phase II). In Phase I, confusion among residents, medical institutions and PHCs due to the unknown virus overwhelmed PHNs. In Phase II, PHNs faced difficulties, such as the inability to control the pandemic, uncooperative patients with prevention control and an unsustainable organizational environment. With such challenges, PHNs experienced emotions such as physical and mental exhaustion, distress, fear, nervousness, conflict and dissatisfaction. Their distress in terms of the responsibility of saving residents' lives and having an identity crisis, which are peculiar to specialized medical personnel, accumulated through all phases of the pandemic.

#### 3.2.1. Overwhelmed with confusion by the unknown virus

Distress among PHNs, particularly in the early phase, was due to the uncertainty of the SARS-CoV-2 related disease. PHNs, who were the only contact points in the community, became one-sided outlets of complaints and outbursts from people who panicked with fear and anxiety about the new threat of the unknown virus. In addition, these complaints were greatly influenced by their misinterpretation of media reports that did not faithfully reflect the current situation of PHCs and the healthcare system. Under such circumstances, PHNs were at the mercy of the residents' verbal abuse and faced difficulty ensuring that residents understood appropriate information.

At that time, gaining cooperation from local medical institutions to triage and detect infected individuals became an urgent issue for PHCs. However, as healthcare workers in medical institutions were also panicked, many physicians and medical institutions refused to take risks and responsibility, and few cooperated with PHCs. PHNs faced difficulty in finding medical resources and responding to triage judgments left by local physicians. Moreover, they struggled with having to respond to local physicians complaining that PHCs were recommending that residents with symptoms of the common colds visit them for consultation.

In Phase I, inquiries from residents flooded the PHCs, since it had been the only local consultation service officially designated by the Ministry of Health, Labor and Welfare. Although little knowledge of the virus was available in the early phase, PHNs were asked for more detailed information. With few medical resources to provide tests for suspected patients, PHNs had no choice but to refuse test requests from residents. Moreover, these situations created a large gap between people's expectations and what PHCs could offer, making residents and medical institutions angry and overwhelming the PHNs.

#### 3.2.2. Inability to control the pandemic

PHCs are responsible for the active epidemiological investigation, giving health guidance for prevention, medical coordination and follow-up during home treatment for all patients. They have a responsibility to prevent the spread of infection and for fair distribution of medical care in the community. These responsibilities are stipulated in the Japanese Infectious Disease Control Law. The active epidemiological investigation is generally conducted immediately upon a ‘notification of the occurrence of a positive case’ from medical institutions to triage and coordinate medical facilities promptly.

PHNs were exhausted and disappointed by the continuous uncertainty that they had been facing for hours, months or years, wondering when the number of confirmed cases would peak, how many confirmed cases would be reported that day, the time to leave work after completing investigations and medical coordination, and the duration of such situations. Because the virus was invisible and constantly mutating, guessing the trend of confirmed cases was not easy. In addition, due to the PHC's function, there was no limit to the number of ‘notifications’ from local medical institutions, unlike hospitals with a concept of “full beds”.

However, there was anguish not only when the number of confirmed cases increased, but also when the wave was on a downward trend. Some PHNs expecting the next wave felt a sense of aversion, saying, “I do not want to imagine the next wave anymore”, considering the past traumatic and distressing experience of dealing with many positive cases. Regardless of such feelings, they could not release tension to prepare for the next wave, such as holding infection control training sessions for nursing care professionals in welfare facilities and developing organizational systems. We clarified the peculiarity of the pandemic, including the repeated peaks, uncontrollability and the uncertainty of the wave's timing, that directly affected their work and exhausted them.

#### 3.2.3. Uncooperative patients with prevention control

PHNs cannot prevent infection in the community and protect patients' lives without the understanding and cooperation of the community, including medical institutions and community residents. Specifically, PHNs have been requesting patients to refrain from working or going out, close contacts to have tests and stay home and the general population to take general infection prevention measures such as keeping their distance and wearing masks. They have been working to protect the residents and the community.

However, PHNs' intentions and thoughts were not appropriately understood in the community due to the residents' excessive personal emotions, such as aversion to restrictions on behavior, and cultural differences. Furthermore, PHNs were distressed by becoming the outlet for unreasonable demands, verbal abuse, complaints, one-sided feelings due to excessive emotions, a sense of entitlement from paying taxes and dissatisfaction with services provided by PHCs. At the same time, there was a sense of impatience; complaints from residents slowed down other duties, including medical coordination for patients needing medical care and first contact for the huge volume of diagnosed patients.

Until the end of the fifth wave, i.e., in December 2021, PHNs had requested those who had close contact with a COVID-19 case to stay home for 14 days, which was longer than the 10 days for positive cases, following the national policy. However, it was without any legal basis. In Japan, unlike patients whose lives during quarantine were guaranteed by law, there were no such restrictions and guaranty for close contacts. Considering the position of those who had close contact with a positive case, PHNs had conflicts in fulfilling their role of requesting cooperation to prevent infection spread, even though they understood the complaints of long restrictions without any support.

#### 3.2.4. Unsustainable organizational environment

They were frustrated with their inability to effectively play their roles as professionals working at the frontline in the community, with delays in organizational arrangements, insufficient instructions and a lack of time for staff members to reach a consensus and resolve their concerns.

In the recent pandemic, even though it was the unprecedented emergency at the level of disaster, a clear division of roles throughout the PHC was not instituted. This promoted staff from other divisions to “assist” PHNs rather than act independently. Another issue was the delay and inconsistency in system development, including data management, readiness to receive support staff and building a unified flow appropriate for practice setting within the organization. Clear directions by leaders, such as designating a stopping point for their duty in each day, were also required. These delays and ambiguous development of organizational systems made it impossible to adequately perform the responsibilities and functions as PHNs and PHCs. Because the organization would not change, despite its capacity, frustrations ensued. Even when the situation seemed to be improved, new issues emerged. For example, when the sharing roles were promoted by dispatching staff from outside PHCs, PHNs struggled with instructing and communicating information to newly dispatched personnel each day and unifying the responses of support staff.

There were also difficulties related to the consistency of recognition within the organization and problem-solving process that could not be changed with the development of the organizational structure. As the number of confirmed cases increased, all staff members were occupied with their duties of communicating with residents over the phone, making it difficult to interact with each other. As a result, PHNs found it difficult to resolve their concerns and to express frank dissatisfaction and opinions with the organizational structure, which are frequently considered as less urgent and low priority. This delayed further organizational structure development and improvements in operational efficiency and hindered uncertainty elimination in responses.

#### 3.2.5. Accumulating liability to save residents' lives under limited medical resources

In Japan, examination, testing, diagnosis and prescription of suspected or confirmed cases are conducted at local hospitals and clinics. In most cases, PHCs do not diagnose or prescribe, but conduct quarantine and medical coordination activities after they receive notifications from local physicians. PHCs have played an essential role in the allocation of medical resources, particularly in regard to testing in the early phase of the pandemic and medical facilities for inpatients and home care patients as the number of confirmed cases increased. However, the critical role of saving residents' lives cannot be fulfilled without the cooperation of local medical institutions. The number of cooperative medical institutions admitting and receiving suspected/positive patients was quite limited because of their staff levels, capabilities and facilities.

Therefore, as the number of confirmed cases and patients needing medical care increased, PHNs felt huge anxiety about their inability to allocate medical resources and save patients' lives. PHNs were frustrated with being caught between patients and medical institutions. It was an important role of PHNs to advocate for patients in need of medical care. However, they felt distressed when they received further requests of patients to be hospitalized during a crisis in which patients who already needed to be hospitalized were instead being treated at home.

They also felt pressure to make decisions quickly, based on information they received through phone communication, amid the demand for fair medical allocation. Some PHNs were pointed out triage errors by physicians when they had directed non-urgent patients to medical institutions.

PHNs in some PHCs were exhausted because of extra duties, such as taking emergency cell phones home to respond to sudden changes in home care patients at night, requesting further cooperation from medical institutions in response to a lack of medical resources and operating testing centers on behalf of medical institutions.

#### 3.2.6. Identity crisis due to inability to carry out the PHN's role of controlling infection in the community

With increased numbers of confirmed cases, the need to collaborate with local healthcare professionals and share personal medical information increased. Thus, PHNs were forced to ask other professionals to provide the first contact with the target population, such as active epidemiological investigation and guidance on infection prevention to those in-home care and close contacts, which had been the main responsibility of PHNs until then. Some PHNs perceived prevention of the spread of infection throughout the community as paramount in their duties as PHNs. Those with such identities felt conflict about not fulfilling their role while recognizing the importance of coordinating medical resources for individual positive patients in need of medical care.

## 4. Discussion

This study reveals the distress and emotions of PHNs in having to confront a situation they had never experienced before. It goes without mentioning the need for social and mental support, considering the difficulties they have experienced, their emotions and distress. We examined their distress from four perspectives: crisis communication, resilient organization, heavy liability and identity crisis.

### 4.1. Crisis communication: Communication among PHC and residents in a health crisis

The PHNs were exhausted from dealing with the complaints and verbal abuse from confused residents and medical institutions, especially in the early phase. They were also frustrated by the difficulty of gaining understanding of PHC responses and requests for cooperation from the community.

During this pandemic, the whole world was concerned about an unknown and invisible threat, and people were strongly affected by negative emotions such as fear, anxiety and anger [25]–[27]. With limited information released by the government due to insufficient knowledge and protecting human rights, people actively sought information to get a clearer picture of the situation. Comparing January 2020 and April 2020, when the state of emergency was declared for the first time, the number of searches for ‘COVID-19’ on the internet increased about 100 times in Japan. TV viewing time increased about 1.3 times, especially news programs [28].

Originators of mass media and social media tend to disseminate false or exaggerated information to respond to the needs of people in such crises, and confusion reigned [29]. During the pandemic, PHNs were forced to clear up people's misunderstandings, probably caused by the mass media's dissemination of information that differed from reality. In the case of the Wakayama poisoned curry incident in 1998, no matter how many explanations were given by the PHC, residents did not listen to them because people gave more credit to misleading information from the media [30]. In addition, according to Kasperson et al. [31], even if the amount of information is equal during a crisis, positive information cannot have a greater impact on the recipients than negative information, which increases risk perception. Moreover, during the Ebola outbreak in the USA, negative information was used more frequently in news reports than positive information, such as the infection being preventable [32]. Another characteristic of the social context of this pandemic is that the development of the information society further accelerated [33],[34] and broadened the spread of rumors and information with varying degrees of credibility. Even in 2014, during the Ebola outbreak in the USA, about 10% of the related tweets contained false information, with the most common content related to government conspiracies [35].

PHCs were the only contact points in the community where people, whose anxiety was heightened by the various false and exaggerated information and rumors, could express their emotions. In past health crises, local government officials, including PHNs, responded to residents' complaints and stress [12],[36],[37]. PHNs became emotional targets of residents misled by rumors [38]. Since normal times, PHNs in PHCs have supported residents, patients and their families in cases such as intractable diseases and mental health. They are responsible for coordinating social resources according to the situation. However, the contact point that was supposed to provide such consultation services for COVID-19 became the target of one-sided abuse and complaints; this was consistent with the results of Usukura et al. [14]. Despite providing a consultation service, PHNs would have experienced psychological distress and an identity crisis when responding to people's complaints and verbal abuse [39].

In response to this, it is necessary to re-examine the dissemination of information by the sources, especially mass media, during crises. At the same time, measures are needed to improve the health literacy and media literacy of the recipients [29]. It is also necessary to assess crisis communication by PHCs, including how PHCs provide information that encourages the community to take appropriate action.

Crisis communication is defined as all communication activities when an organization, such as a company or government agency, is in a crisis due to an incident or accident; it is mainly aimed at protecting the organization from secondary damage caused by rumors and overcoming the crisis to achieve sustainable growth [40]. Our study revealed that PHNs struggled to have interactive communication with residents whose views were one-sided, even though they had been communicating one-on-one by telephone. Many municipalities have centralized information dissemination on health crises at their main governmental offices in Japan. According to Su et al., during this pandemic, the lack of data, evidence, consensus about COVID-19 and a best approach to control the pandemic and the ever-evolving nature of COVID-19 made it difficult to deliver appropriate information [41]. However, to protect organizations from secondary damage and increase sustainability in the future, it will be necessary for PHCs to utilize social media to increase the dissemination of reliable information. They should focus on the needs of residents and the current situation in specific areas to achieve interactive communication.

In general, the intention and purpose of the PHNs' activities and the PHCs' roles are difficult to be understood by the community [42],[43]. Therefore, disseminating information on the role, the services and the current situation within PHCs are required to ensure the transparency of their activities. In addition, Ratzan et al. [29] stated that the initial rumors should be picked up to prevent the spread of false information. Disseminating information in response to the demands of residents and medical institutions would prevent the spread of incorrect information and support gathering reliable information. For example, this could include frequently asked questions and answers, facts about the virus, what is already revealed and what is still yet unknown and the flow for those diagnosed positive and close contacts. Promoting people's understanding through the dissemination may enable PHCs to implement measures that should be prioritized for that phase efficiently.

### 4.2. Resilient organization in a pandemic

Japanese PHNs involved in the flood disaster in 2009 reported anxiety about the unpredictable situation [13]. Its long duration and repeated waves, which grow larger and larger each time, is the feature of the current pandemic. Considering the characteristics, it is expected that PHNs may have faced great suffering that has not been experienced in previous health crises, including pandemics such as influenza H1N1 and severe acute respiratory syndrome (SARS) in Japan. In addition, in the previous natural and nuclear disasters in Japan, PHCs have responded by suspending normal operations, switching to a crisis management system immediately after a disaster and dividing roles within the PHCs [30]. However, the PHCs were conservative during the current pandemic; PHNs described the pandemic as a “disaster level”, “long-term” and “unpredictable situation”. However, the development of a sustainable organizational structure and provision of appropriate instructions commensurate with the situation were insufficient.

According to research by Tomioka et al. [44], the number of confirmed cases was high in prefectures with a small number of PHNs per population during this pandemic. This indicates the effectiveness of the active epidemiological investigation and other health services relating to COVID-19 provided by PHNs in the community. Creating a sustainable organization through flexible reforms according to such characteristics of the pandemic are critical because it would promote their work efficiency and may impact the protection of the community. To achieve a resilient community against the pandemic and any other health crisis, rebuilding a resilient organizational structure of PHCs is an urgent issue.

### 4.3. PHN's liability and lack of medical resources in the pandemic

Japanese PHCs are responsible for coordinating medical institutions for patients who require hospitalization or medical consultation. As the only specialized organizations in the community, they were burdened with a heavy responsibility to make decisions, including those relating to life-threatening matters. Our study revealed that, despite the life-threatening crisis and residents seeking help, PHNs felt a sense of hopelessness, anxiety and conflict because they could not adequately protect people's lives.

One of the reasons for their liability was a shortage of distributable medical resources. Since PHCs do not conduct medical examinations as hospitals and clinics, gaining the cooperation of local medical institutions was needed to protect the lives and prevent the spread of infection. However, there was not enough cooperation due to the special circumstances of the pandemic and lack of preparation in advance. Moreover, PHNs also suffered from complaints and intimidating claims from medical staff. Insufficient knowledge of the virus may have caused medical resource shortages. According to Almohammed et al. [45], adequate knowledge about COVID-19 was related to positive attitudes toward the pandemic, including the fact that ‘all people in the healthcare system should be responsible about their role’. It can be inferred that factors such as uncertainty of the SARS-CoV-2 related disease, risk of infection and unfamiliarity with care inhibited cooperation, since healthcare providers should have direct contact with positive confirmed individuals. Tanaka et al. reported that medical coordinators in the Great East Japan Earthquake could accommodate most of the injured and sick because they had established a face-to-face relationship with the medical institutions daily in advance [46]. In our interviews, one PHN stated that “coordination goes more smoothly with PHNs who have a face-to-face relationship with the nurse in hospitals”. This also indicates PHCs' need to regularly visit local hospitals and clinics to build relationships and disseminate information, and to encourage local healthcare workers to raise their awareness of infection control.

In addition, they also described the difficulty of collecting accurate information from patients over the phone and properly assessing illness severity. One study revealed that patient triage was one of the physician's stressors during the COVID-19 pandemic [47], and nurses who perform triage over the phone as daily duty had difficulty determining the urgency and severity of cases [48],[49]. Since PHNs have little experience with triage in their daily work, their anguish and distress in being forced to assume the same role as triage nurses with limited medical resources was immeasurable. It may be necessary to reevaluate information transmission methods in order to achieve more efficient and accurate triage, such as by replacing the phone with a device that enables information-sharing through multi-party communication and video calls.

### 4.4. Identity crisis

PHNs who work as civil servants are sometimes described as “men of all work” [39] due to their complex position as administrative and medical personnel. Moreover, PHNs take on “a job that is ultimately anyone's job” [43], which is different from their primary duty as PHNs, making them vulnerable to occupational identity crises [50].

In the current crisis, PHNs played more roles in protecting individuals' lives instead of preventing infection in the community, and they responded to people's complaints and verbal abuse instead of consultations. These gaps were because of the huge number of confirmed cases, the needs of, and roles expected by, residents, and the limited work that a PHN can practically do. Since they are always aware of individual and community perspectives in their health activities, each PHN's perception of their role may differ. However, some PHNs were found to have a strong sense of mission to protect the community more than individuals, and they strongly perceived this as their own identity. In Japan, PHNs are the only ones who conduct health activities targeting the community as a group, while general nurses in medical institutions can provide care for individuals. This particularity of their job may create their identity as a PHN.

Shoji et al. [51] stated that the PHN's professional identity is formed by pride in their awareness as a PHN and their experiences of overcoming difficulties while working with diverse people. Posting other professionals in a division together and enabling PHNs to focus on their primary duties can maintain and improve their identity recognition. In future pandemics, especially in the early phase, there is expected to be a great deal of confusion among residents and medical institutions. In particular, in responding to a pandemic in which PHNs will play a central role, it is necessary to divide the tasks that anyone can do and those that only PHNs can do. Moreover, unlike this time during which the organizational setup including personnel distribution was not performed smoothly, assignment of staff, increased numbers of PHNs and division of the roles among them from the initial phase are required. It will also be necessary to develop hardware, such as an automatic voice guidance system that can direct inquiries from community residents to the appropriate professionals in PHCs. This would allow more time to setup a PHC organization to better respond to health crises, strengthening cooperation through careful coordination with medical institutions and providing health guidance to positive patients or close contacts. This will help maintain organizational sustainability and achieve more effective quarantine measures, even for prolonged conditions.

### 4.5. Limitations

In our study, qualitative investigations were conducted with only 12 PHNs in PHCs belonging to one prefecture in Japan. The challenges and distresses faced by PHNs may differ between PHCs in other regions. Therefore, the findings cannot be directly applied to all organizations and PHNs due to different circumstances and situations. In the future, it will be necessary to expand the scope of the study to include other regions and target populations.

## 5. Conclusions

There was remarkable anguish experienced by PHNs in Prefecture A at the frontlines of quarantine in a pandemic that occurred amid a reduction in the number of PHCs in Japan. The PHNs were aware of the fear and anxiety of the general population against the unknown infectious disease, and, even with limited medical resources, they tried to fulfill their role as nurses to advocate for residents in the community. There was also a unique awareness among Japanese PHNs to protect individuals and the entire community by reaching out to residents. However, it became clear that the environment in which PHNs could effectively fulfill their roles and contribute to community infection control had not yet been established at PHCs at regional, prefectural and national levels. Although 3 years of quarantine activities have helped to adapt to this situation, the COVID-19 pandemic is still ongoing. As PHNs continue to fulfill their professional roles in this pandemic, they will continue to experience psychological distress. It is expected that their distress will continue in the long term and during future health crises, such as another pandemic or disaster. Therefore, future roles of PHNs in health crises need to include strengthening local public health systems and interacting with residents and medical institutions as community coordinators.

## References

[1] WHO (2022). WHO coronavirus (COVID-19) dashboard.

[2] Ministry of Health, Labor and Welfare (2002). Health crisis management in the community-guidelines for community health crisis management. Japan Ministry of Health, Labor and Welfare.

[3] National Association of Public Health Center Directors (2021). Changes in the number of public health centers. Japan National Association of Public Health Center Directors.

[4] Ogata T (2021). COVID-19 up-to-date, role and challenges of public health center in response to COVID-19. Modern Media.

[5] Osumi M (2021). Rise in COVID-19 cases across Japan takes toll on public health centers. The Japan Times.

[6] Kyodo (2022). 20% of Japan's public health center staff overworked with COVID-19 duties. The Japan times.

[7] Ide K, Asami T, Suda A (2021). The psychological effects of COVID-19 on hospital workers at the beginning of the outbreak with a large disease cluster on the Diamond Princess cruise ship. PLoS One.

[8] Lai J, Ma S, Wang Y (2020). Factors associated with mental health outcomes among health care workers exposed to coronavirus disease 2019. JAMA Netw Open.

[9] Sasaki N, Kuroda R, Tsuno K (2020). The deterioration of mental health among healthcare workers during the COVID-19 outbreak: A population-based cohort study of workers in Japan. Scand J Work Environ Health.

[10] Stone KW, Kintziger KW, Jagger MA (2021). Public health workforce burnout in the COVID-19 response in the U.S.. Int J Environ Res Public Health.

[11] Du Z, You H, Zhou H (2021). Difficulties encountered by public health workers in COVID-19 outbreak: a cross-sectional study based on five provinces. BMC Health Serv Res.

[12] Yamada H, Kusumi M, Yoshida H (2013). The research on the mental health and body health condition toward public health nurses working for the victims of east Japan great earthquake. Jpn Soc Health Sci Mind Body.

[13] Ushio Y, Osawa T, Shimizu M (2012). The psychological influence of a disaster experience on municipal staff; interviews with public health nurses sixteen months after a flood disaster. UH CNAS, RINCPC Bulletin.

[14] Usukura H, Seto M, Kunii Y (2021). The mental health problems of public health center staff during the COVID-19 pandemic in Japan. Asian J Psychiatr.

[15] Nishimura Y, Miyoshi T, Hagiya H (2022). Prevalence of psychological distress on public health officials amid COVID-19 pandemic. Asian J Psychiatr.

[16] Sandelowski M (2000). Whatever happened to qualitative description?. Res Nurs Health.

[17] Neergaard MA, Olesen F, Andersen RS (2009). Qualitative description-the poor cousin of health research?. BMC Med Res Methodol.

[18] Spencer RA, Joshi N, Branje K (2021). Early childhood educator perceptions of risky play in an outdoor loose parts intervention. AIMS Public Health.

[19] Knott A, Kee CC (2005). Nurses' beliefs about family presence during resuscitation. Appl Nurs Res.

[20] Krippendorff K (2004). Content Analysis: An Introduction to Its Methodology.

[21] Graneheim UH, Lindgren B, Lundman B (2017). Methodological challenges in qualitative content analysis: A discussion paper. Nurse Educ Today.

[22] Graneheim UH, Lundman B (2004). Qualitative content analysis in nursing research: concepts, procedures and measures to achieve trustworthiness. Nurse Educ Today.

[23] Lincoln YS, Guba EG (1985). Naturalistic inquiry.

[24] Guba EG, Lincoln YS (1989). Fourth Generation Evaluation.

[25] Goularte JF, Serafim SD, Colombo R (2021). COVID-19 and mental health in Brazil: psychiatric symptoms in the general population. J Psychiatr Res.

[26] Maggi G, Baldassarre I, Barbaro A (2021). Mental health status of Italian elderly subjects during and after quarantine for the COVID-19 pandemic: a cross-sectional and longitudinal study. Psychogeriatrics.

[27] Midorikawa H, Aiba M, Lebowitz A (2021). Confirming validity of the fear of COVID-19 Scale in Japanese with a nationwide large-scale sample. PloS One.

[28] Intage (2020). Media contact behavior change during COVID-19 pandemic, Capturing the changes after GW week and after lifting of the state of emergency declaration. Japan Intage.

[29] Ratzan SC, Sommariva S, Rauh L (2020). Enhancing global health communication during a crisis: lessons from the COVID-19 pandemic. Public health Res Pract.

[30] Tatara K, Takatorige T, Kondo T (2002). Promoting health crisis management in the community: toward specific counter-terrorism measures, Shinkikaku Shuppan.

[31] Kasperson RE, Renn O, Slovic P (1988). The social amplification of risk: A conceptual framework. Risk Analysis.

[32] Sell TK, Boddie C, McGinty EE (2017). Media messages and perception of risk for Ebola virus infection, United States. Emerg Infect Dis.

[33] Ministry of International Affairs and Communications (2021). WHITE PAPER Information and Communications in Japan. Japan Ministry of International Affairs and Communications.

[34] Eurostat (2022). Individuals using the internet for seeking health-related information. European Union Eurostat.

[35] Sell TK, Hosangadi D, Trotochaud M (2020). Misinformation and the US Ebola communication crisis: analyzing the veracity and content of social media messages related to a fear-inducing infectious disease outbreak. BMC Public Health.

[36] Suzuki Y, Fukasawa M, Obara A (2014). Mental health distress and related factors among prefectural public servants seven months after the great east Japan earthquake. J Epidemiol.

[37] Suzuki Y, Fukasawa M, Obara A (2017). Burnout among public servants after the Great East Japan Earthquake: decomposing the construct aftermath of disaster. J Occup Health.

[38] Kayama M, Akiyama T, Ohashi A (2014). Experiences of municipal public health nurses following Japan's earthquake, tsunami, and nuclear disaster. Public Health Nurs.

[39] Saeki K (2022). What is the power of continuing to work as a public health nurse? What it means to work as a public health nurse in a changing business and society. Jpn J Public Health Nurs.

[40] Uozaki H (2010). Crisis Communications for Confidence and Safety. J Jpn Soc for Safety Eng.

[41] Su Z, Zhang H, McDonnell D (2022). Crisis communication strategies for health officials. Front Public Health.

[42] Murai F, Yasuda T (2016). The current situation regarding support provided by public health center nurses in response to infectious disease: outbreaks at facilities for the elderly. Bulletin Nagano College of Nursing.

[43] Tsuboi R, Iida M, Osawa M (2013). Dilemmas of municipal public health nurses in the welfare sector who provide individual support to psychiatric patients: elements of dilemmas and their relationships. J Jpn Acad Community Health Nurs.

[44] Tomioka K, Shima M, Saeki K (2022). Number of public health nurses and COVID-19 incidence rate by variant type: an ecological study of 47 prefectures in Japan. Environ Health Prev Med.

[45] Almohammed OA, Aldwihi LA, Alragas AM (2021). Knowledge, attitude, and practices associated with COVID-19 among healthcare workers in hospitals: a cross-sectional study in Saudi Arabia. Front Public Health.

[46] Tanaka K, Yamada Y, Fukuda K (2014). An experience report in the transport hub for the region in the acceptance of the great east Japan earthquake. Jpn J Disaster Med.

[47] Lamiani G, Biscardi D, Meyer EC (2021). Moral distress trajectories of physicians 1 year after the COVID-19 outbreak: a grounded theory study. Int J Environ Res Public Health.

[48] Holmström I, Höglund AT (2007). The faceless encounter: ethical dilemmas in telephone nursing. J Clin Nurs.

[49] Holmström IK, Kaminsky E, Lindberg Y (2021). The perspectives of Swedish registered nurses about managing difficult calls to emergency medical dispatch centres: a qualitative descriptive study. BMC Nurs.

[50] Saeki K (2012). Considering the “sense of mission” of public health nurses: the soul of public health nurses today. Jpn J Community Health Care.

[51] Shoji H, Ueno M, Okawa S (2018). Factors influencing the formation of professional identity from municipal health nurse's experience process-narrative of experienced public health nurse's experience. Bull Fac Nurs Kobe Women's Univ.

